# Mechanism of the AC-Light-Shift-Induced Phase Shift and a DC Compensation Strategy in Bell–Bloom Magnetometers

**DOI:** 10.3390/s25226871

**Published:** 2025-11-10

**Authors:** Rui Zhang

**Affiliations:** National Innovation Institute of Defense Technology, AMS, Beijing 100071, China; dr.ruizhang@163.com

**Keywords:** AC light shift, phase shift, atomic magnetometer, heading error, compensation

## Abstract

The Bell–Bloom magnetometer is promising for mobile applications, but its accuracy is limited by heading errors. A recently identified source of such error is a phase shift in the magnetic resonance, which arises from the superposition of two signals, i.e., the primary resonance from synchronous pumping and a secondary resonance, 90° out-of-phase, driven by the AC light shift of the pump laser. Through Bloch equation modeling and experiment, we uncover a counter-intuitive mechanism: although initiated by the AC light shift, the phase shift’s magnitude is determined solely by the pump light’s average power (DC component) and is independent of its AC modulation. This occurs because the amplitude ratio of the two resonances depends exclusively on the DC-power-induced atomic polarization. Based on this insight, we propose a novel DC compensation scheme by adding a continuous counter-polarized beam to cancel the net DC pumping. Theoretically, this simultaneously suppresses both the AC-light-shift-induced phase shift and the DC-light-shift-induced frequency shift. The scheme’s advantage is its simplified approach to polarization control, avoiding the need for high-speed polarization modulation or major hardware changes as the beams share the same optical path. This makes it highly suitable for demanding mobile applications like aerial magnetic surveying and wearable bio-magnetic sensing in unshielded environments.

## 1. Introduction

Atomic magnetometers have attracted significant interest due to their high sensitivity, room-temperature operation, and potential for miniaturization, making them suitable for a wide range of applications such as geological exploration [1,2,3,4] and bio-magnetic sensing [5,6]. Unlike vector magnetometers, which measure individual field components, scalar atomic magnetometers theoretically detect the magnitude of the magnetic field [7]. This property makes them inherently less sensitive to the orientation of the sensor. This theoretical orientation-independence is particularly advantageous on moving platforms, for instance, in airborne surveys [2,8] or wearable devices [6], where the orientation changes continuously. In the presence of a large ambient field such as the Earth’s magnetic field (∼25 to 65 µT [9]), even small misalignments or non-orthogonalities in vector sensors can lead to significant measurement errors (e.g., an error of tens of nT for a 1 mrad orientation error) that require careful calibration [10,11]. In contrast, scalar magnetometers are expected to be free of such issues.

In practice, however, atomic magnetometers still exhibit residual heading errors on the order of nanotesla (nT). This means the measured field value changes as the sensor is rotated, even when the actual field remains constant [7]. Although these errors are generally smaller than those of uncalibrated vector sensors in large background fields, they remain much larger than many target signals of interest, which are often at the picotesla (pT) level. Thus, heading error represents a critical obstacle for high-precision applications. Several physical mechanisms are known to contribute to this effect, including the nonlinear Zeeman effect [12,13,14], nuclear Zeeman effect [14,15], and DC light shift [13]. More recently, it has been discovered that in Bell–Bloom magnetometers using circularly polarized pump light, an additional heading error can arise from the AC component of the light shift [16].

In such magnetometers, the modulated pump beam does more than just polarize the atoms; it also creates an oscillating “fictitious magnetic field” through the AC light shift, which points along the pump beam’s propagation direction. Although this field oscillates rapidly, its interaction with the atomic spins is not averaged to zero. When the pump modulation is synchronized with the Larmor precession, this oscillating fictitious field can resonantly drive the spins. Crucially, this interaction generates a secondary magnetic resonance signal. This secondary signal is naturally shifted by 90° relative to the primary resonance signal produced by synchronous optical pumping. The vector addition of these two signals results in a net phase shift of the combined resonance signal. Since the orientation of the fictitious field is tied to the pump beam, this phase shift becomes orientation-dependent. Consequently, when using phase-sensitive detection methods common in these sensors, this manifests as a heading error [16]. To suppress this effect, one previously proposed method incorporates a half-wave plate inside the vapor cell to reverse the pump polarization, thereby averaging out the fictitious field effects over the cell length [16]. Theoretically, a push–pull pumping scheme [17], which reverses the circular polarization of the pump light within each modulation cycle, could also average out this effect temporally.

In this work, we investigate the phase shift induced by AC light shift in a Bell–Bloom magnetometer. Counter-intuitively, we find that this phase shift depends not on the AC modulation amplitude, but on the DC component of the pump power. Theoretical analysis based on the Bloch equations indicates that the DC component contributes not through its associated DC light shift, but through the atomic polarization it creates, which then interacts with the AC light shift to produce the phase shift. We experimentally confirm that the phase shift is correlated to the DC pump power and independent of the AC modulation amplitude. Based on this understanding, we propose a novel suppression scheme: adding a continuous counter-polarized beam to cancel the atomic polarization induced by the DC pump component. This approach eliminates the phase shift and the DC light shift, without requiring polarization reversal or active, high-speed polarization modulation of the main pump beam, offering a potentially more straightforward experimental implementation in terms of polarization control. Collectively, the physical picture and the compensation strategy established in this work are completely different from those of the geometric phase study in our prior work [18].

## 2. Experiment  Setup

The core configuration of the Bell–Bloom magnetometer used in this work is similar to those detailed in Refs. [18,19]. It employs a circularly polarized pump beam to polarize the atoms and a linearly polarized probe beam to monitor the spin evolution. The pump beam is intensity-modulated near the Larmor frequency to synchronize the atomic precession. The optical rotation angle of the probe beam is proportional to the atomic magnetization component along its propagation path.

A 2 cm square cesium vapor cell, containing 300 Torr of helium and 25 Torr of nitrogen buffer gas, was operated at room temperature. The pump laser was frequency-locked to the Cs D1 transition from the ground state F=3 to the excited state $F^{\prime }=3$ (wavelength ∼ 894.6 nm). This configuration enables spin polarization on the ground state F=4 via indirect optical pumping [20,21], which helps mitigate power broadening. The pump beam was modulated with a 50% duty cycle using an acousto-optic modulator, with the modulation frequency set close to the Larmor frequency and its power adjusted as needed. The probe laser was locked to the Cs D2 transition from F=3 to $F^{\prime }=4$ (wavelength ∼ 852.3 nm), allowing effective detection of the F=4 ground-state spin evolution via optical rotation while minimizing interference from the F=3 state. The probe power was maintained at 40.8 µW. Both beams were delivered to the vapor cell via separate fiber collimators (output diameter ~ 2.6 mm), arranged to intersect perpendicularly within the cell. The optical rotation of the probe beam was detected using a polarimeter comprising a polarizing beam splitter and a balanced photodetector. The resulting signal was demodulated by a lock-in amplifier to extract the magnetic resonance signal. Further details on the fundamental operating principles are available in the supplementary materials of ref. [19].

**Figure 1:**
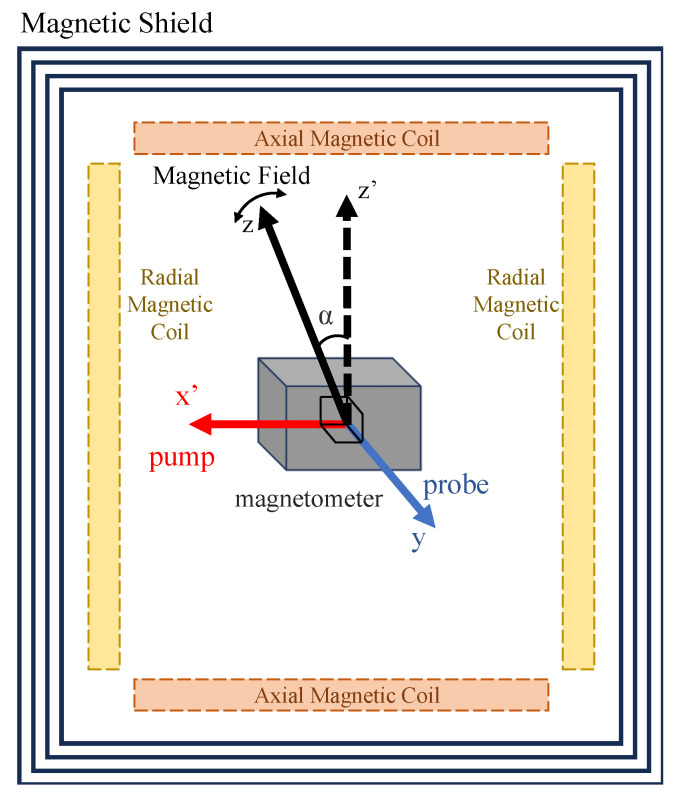
Schematic of the experimental setup for controlling the angle between the pump beam and the magnetic field. The atomic magnetometer is housed within a multilayer magnetic shield, with both the pump (red arrow) and probe (blue arrow) beams oriented perpendicular to the shield’s axial direction ($z^{\prime }$-axis, black dashed arrow). The direction of the background magnetic field (*z*-axis, black solid arrow) within the $x^{\prime }$-$z^{\prime }$ plane is controlled by adjusting the currents in a pair of orthogonal uniform magnetic field coils, thereby varying the angle relative to the fixed pump beam.

To investigate the AC-light-shift-induced phase shift, which depends on the angle between the pump beam and the background magnetic field, this angle was systematically varied. As shown in Figure 1, the magnetometer was housed inside a multilayer magnetic shield, with the plane defined by the pump and probe beams aligned with the shield’s transverse cross-section. The pump beam propagated along the $x^{\prime }$-axis, the probe beam along the *y*-axis, and the shield’s cylindrical axis defined the $z^{\prime }$-axis. The direction of the background magnetic field within the $x^{\prime }$-$z^{\prime }$ plane was precisely controlled using a pair of orthogonal uniform magnetic field coils. An axial coil generated a field along the $z^{\prime }$-axis (the cylindrical axis of the magnetic shield, indicated by the vertical black dashed arrow in Figure 1), while a radial coil generated a field along the $x^{\prime }$-axis (aligned with the pump beam propagation direction). By adjusting the current ratio in these two coils, the resultant magnetic field vector was rotated from the $z^{\prime }$-axis towards the pump direction ($x^{\prime }$-axis) by an angle α. For theoretical consistency, the direction of the background magnetic field is consistently defined as the *z*-axis in our model (indicated by the solid black arrow in Figure 1), regardless of its spatial orientation. The background field magnitude was maintained at approximately 1000 nT. The remnant field inside the magnetic shield, measured by a three-axis fluxgate sensor, was below 10 nT in all directions. No external field was applied along the *y*-axis (perpendicular to the plane of the diagram). Thus, the field’s strength and orientation were precisely controlled by the coils, confining the net magnetic field vector to the plane parallel to the diagram. For each experimental configuration, the angle α between the background field and the $z^{\prime }$-axis was set to the desired value.

## 3. Theory

### 3.1. Introduction of the Bloch Equation Model

The spin dynamics are modeled by a Bloch equation (Equation (1)), which is adapted from our previous work [18] with a modification to include the light shift effect:

(1) $$\begin{array}{c} \frac{dM}{dt}=-\gamma \left(B_{0}+B_{\mathrm{LS}}\right)\times M-\left(\Gamma_{2}M_{x}\hat{e_{x}}+\Gamma_{2}M_{y}\hat{e_{y}}+\Gamma_{1}M_{z}\hat{e_{z}}\right)-\Gamma_{P}\left(M-M_{0}\right). \end{array}$$

Briefly, the equation describes the time evolution of the magnetic moment vector M under three influences: precession around a magnetic field comprising the external field $B_{0}$ and the effective fictitious field $B_{\mathrm{LS}}$ induced by the light shift; relaxation of the magnetization components at rates $\Gamma_{1}$ and $\Gamma_{2}$; and optical pumping, which drives the system toward the optical pumping target $M_{0}$ at a rate $\Gamma_{P}$ proportional to the pump laser power. The probe beam’s effect is modeled as an additional relaxation channel (included in the $\Gamma_{1,2}$ terms) and is itself modulated by an optical rotation signal proportional to the component of M along its propagation axis ($M_{y}$). We relate the effective fictitious field $B_{\mathrm{LS}}$ to the optical pumping target $M_{0}$, which represents the polarization of the pump beam, and the pump rate $\Gamma_{P}$ through the relation $B_{\mathrm{LS}}=M_{0}\Gamma_{P}\xi$, where ξ is a constant determined by specific experimental conditions such as the atomic structure, buffer gas pressure, and laser frequency. Both $B_{\mathrm{LS}}$ and $M_{0}$ are oriented along the pump beam’s propagation direction, and their magnitudes are set by the pump beam’s polarization. Furthermore, the strength of $B_{\mathrm{LS}}$ grows linearly with $\Gamma_{P}$.

While our model starts from the Bloch equation framework also used in [18], the introduction of the dynamically modulated light-shift field, $B_{\mathrm{LS}}$, represents a fundamental difference. This addition substantially changes the system’s dynamics by creating a second high-frequency drive, a physical scenario not present in the prior work, which in turn demands a different analytical approach. Consequently, all theoretical development after Equation (1) represents original work tailored to this new problem, where even standard approximations must be applied and justified within a more complex context.

Given that the total magnetic field $B=B_{0}+B_{\mathrm{LS}}=\left(\cos\alpha \cdot M_{0}\xi \Gamma_{P},0,B_{0}+\sin\alpha \cdot M_{0}\xi \Gamma_{P}\right)$ is confined to the *x*-*o*-*z* plane, Equation (1) can be simplified as:

(2a)dMxdt=γBzMy−Γ2Mx−ΓPMx−M0cosα,(2b)dMydt=γMzBx−BzMx−Γ2My−ΓPMy,(2c)dMzdt=−γMyBx−Γ1Mz−ΓPMz−M0sinα,(2d)dM+dt=iγMzBx−iγBz+Γ2+ΓPM++ΓPM0cosα,(2e)dM−dt=−iγMzBx−−iγBz+Γ2+ΓPM−+ΓPM0cosα,

in which $M_{+}=M_{x}+iM_{y}$ and $M_{-}=M_{x}-iM_{y}$.

### 3.2. Steady-State Solution and the Mechanism of the Phase Shift

Our setup employs a square-wave modulation at angular frequency ω for the pump beam. The resulting temporal profile is expressed by the Fourier series

(3) $$\begin{array}{c} \Gamma_{P}=\Gamma \cdot \sum_{n=-\infty }^{\infty }A_{n}e^{in\omega t}, \end{array}$$

where the complex amplitudes $A_{n}$ correspond to each order *n*, and i is the imaginary unit. Through phase control of the modulation, $A_{1}$ is made real. Similarly, we expand M as Fourier series

(4) $$\begin{array}{c} M=\sum_{m=-\infty }^{\infty }M^{\left(m\right)}e^{im\omega t}. \end{array}$$

By substituting Equations (3) and (4) into (2c), (2d) and (2e) and assuming a steady state where $M^{\left(m\right)}$ remains constant, we obtain

(5a)Mz(m)=−γ∑nΓ ⋅ AnMy(m−n)cosα ⋅ M0ξ − ∑n≠0Γ ⋅ AnMz(m−n) + Γ ⋅ AmM0sinαimω + Γ1 + Γ ⋅ A0,iγcosα⋅M0ξΓ⋅∑nAnMz(m−n)                        (5b)M+(m)=−iγsinα ⋅ M0ξ + 1Γ ⋅ ∑n≠0AnM+(m−n) + Γ ⋅ AmM0cosαimω + iγB0 + iγsinα ⋅ M0ξΓA0 + Γ2 + ΓA0,−iγcosα⋅M0ξΓ⋅∑nAnMz(m−n)                       (5c)M−(m)=+iγsinα ⋅ M0ξ − 1Γ ⋅ ∑n≠0AnM−(m−n) + Γ ⋅ AmM0cosαimω − iγB0 − iγsinα ⋅ M0ξΓA0 + Γ2 + ΓA0.

We begin by analyzing Equation (5a), which describes the steady state of $M_{z}^{\left(m\right)}$. This equation expresses the *m*-th Fourier component $M_{z}^{\left(m\right)}$ as a function of other components, such as $M_{y}^{\left(m-n\right)}$ and $M_{z}^{\left(m-n\right)}$. Since both sides of the equation contain unknown variables to be solved for, it is not directly solvable in closed form. However, an approximation can be made by examining the orders of magnitude of the terms on the right-hand side.

The magnitude of the numerator on the right-hand side is determined by terms involving the pumping coefficients $\Gamma A_{n}$ and the optical pumping target $M_{0}$. Although the specific expressions differ for various *m*, a key observation is that each term in the numerator is proportional to either $\Gamma A_{n}M_{0}$ or involves a product of $\Gamma A_{n}$ with a magnetization component (which itself is bounded by $M_{0}$). Consequently, the magnitude of the numerator for any *m* is bounded above by a value on the order of $\max\left(\Gamma_{P}\right)M_{0}$ (where $\Gamma_{P}$ represents the optical pumping rate). This implies that the numerator remains within a similar order of magnitude for all values of *m*, without significant variations that scale with *m*.

In contrast, the denominators of the equation differ significantly. The denominator contains the term imω, where the modulation frequency ω is much larger than the other parameters, such as $\Gamma_{1}$ and $\Gamma A_{0}$. Therefore, for m≠0, the modulus of the denominator, $|im\omega \;+\;\Gamma_{1}\;+\;\Gamma A_{0}|$, is dominated by mω and is much larger than the denominator for m=0, which is $\Gamma_{1}+\Gamma A_{0}$.

Given that the numerator is bounded while the denominator increases substantially with |m|, it follows that for m≠0, the magnitude of $M_{z}^{\left(m\right)}$ is much smaller than $M_{0}$. Thus, a reasonable approximation is to set all higher-order Fourier components $M_{z}^{\left(m\right)}$ (for m≠0) to zero, i.e., $M_{z}^{\left(m\right)}{|}_{m\neq 0}\approx 0$. This approach, which neglects higher-harmonic components, is known as the rotating-wave approximation in physics.

Following the approximation approach applied to Equation (5a), we now analyze Equations (5b) and (5c) for the transverse magnetization components. These equations share a similar mathematical structure with Equation (5a). The magnitude of the numerator on their right-hand sides is similarly bounded above by a value on the order of $\max\left(\Gamma_{P}\right)M_{0}$, while the modulus of the denominator strongly depends on the order *m*. A key difference, however, lies in the denominators of Equations (5b) and (5c), which contain the dominant terms imω and $i(\gamma B_{0}+\gamma \sin\alpha \cdot M_{0}\xi \Gamma A_{0})$. Since the Larmor frequency term $\gamma B_{0}$ and its related component are much larger than the relaxation rates $\Gamma_{2}$ and $\Gamma A_{0}$, and given that the modulation frequency is typically tuned such that $\omega \approx \gamma B_{0}+\gamma \sin\alpha \cdot M_{0}\xi \Gamma A_{0}$, a resonant cancellation occurs in the denominator for specific values of *m*.

Specifically, in Equation (5b), when m=−1, the term imω nearly cancels out the term $i(\gamma B_{0}+\gamma \sin\alpha \cdot M_{0}\xi \Gamma A_{0})$, resulting in a minimal modulus of the denominator. For m≠−1, the denominator’s modulus becomes very large. Similarly, in Equation (5c), the denominator reaches its minimum modulus when m=1, and is very large for m≠1. Given the bounded magnitude of the numerators, significant values for the Fourier components are only expected when the denominator is small. Therefore, for $M_{\pm }^{\left(m\right)}$, all components where m≠∓1 are much smaller than $M_{0}$ and can be approximated as zero, i.e., $M_{+}^{\left(m\right)}{|}_{m\neq -1}\approx 0$ and $M_{-}^{\left(m\right)}{|}_{m\neq 1}\approx 0$. This approximation, consistent with the rotating-wave approximation, retains only the Fourier components that are resonant with the effective driving field.

To obtain explicit expressions for the steady-state magnetization components M, we simplify Equation (5) by employing the three key approximations established, namely $M_{z}^{\left(m\right)}{|}_{m\neq 0}\approx 0$, $M_{+}^{\left(m\right)}{|}_{m\neq -1}\approx 0$, and $M_{-}^{\left(m\right)}{|}_{m\neq 1}\approx 0$. These are combined with the definition $M_{y}^{\left(m\right)}=\frac{i}{2}\left(M_{-}^{\left(m\right)}-M_{+}^{\left(m\right)}\right)$. During the derivation, a first-order approximation was applied because the Larmor frequency from the light-shift field, $\gamma B_{\mathrm{LS}}$, is typically much smaller than the magnetic resonance linewidth, $\Gamma_{2}+\Gamma A_{0}$. Consequently, only the first-order terms in $\gamma B_{\mathrm{LS}}/\left(\Gamma_{2}+\Gamma A_{0}\right)$ were retained, while the higher-order terms were neglected. The simplified results are as follows:

(6a)Mz≈Mz(0)≈M0ΓA0sinαΓ1 + ΓA0,(6b)M+≈M+(−1)e−iωt≈cosα ⋅ M0Γ ⋅ A−1−iδω + Γ2 + ΓA01+iγsinα ⋅ M0ξΓA0Γ1 + ΓA0e−iωt,

in which $\delta \omega =\omega -\gamma (B_{0}+{\bar{B}}_{\mathrm{LS}\|})$, and ${\bar{B}}_{\mathrm{LS}\|}=\sin\alpha \cdot M_{0}\xi \Gamma A_{0}$ represent the DC component of the light-shift field $B_{\mathrm{LS}}$ parallel to $B_{0}$.

The derived steady-state solutions provide insight into the system’s physical behavior. Equation (6a) shows that a stable atomic polarization $M_{z}$ emerges along the magnetic field $B_{0}$ when the pump light is not perpendicular to it (i.e., α≠0°). This polarization is driven by the projection of the average pumping rate onto the field direction, $\Gamma A_{0}\sin\alpha$. Equation (6b) describes the steady-state transverse magnetization $M_{+}$. The time-dependent factor $e^{-i\omega t}$ indicates that $M_{+}$ precesses at the pump modulation frequency ω. Furthermore, the denominator $-i\delta \omega +\Gamma_{2}+\Gamma A_{0}$ reveals a resonance centered at $\gamma (B_{0}+{\bar{B}}_{\mathrm{LS}\|})$ with a linewidth of $\Gamma_{2}+\Gamma A_{0}$. The frequency shift $\gamma {\bar{B}}_{\mathrm{LS}\|}$ originates from the DC component of the light-shift field parallel to $B_{0}$, a well-known effect that causes heading error.

Equation (6b) for the transverse magnetization $M_{+}$ reveals the origin of the anomalous phase shift induced by the AC light shift. The response is not a single resonance but a superposition of two resonances, i.e., the conventional in-phase synchronous pumping resonance and a quadrature resonance induced by the AC light shift. A key, counter-intuitive finding emerges from analyzing their relative amplitudes: although the quadrature resonance is physically caused by the AC light shift, its magnitude relative to the main resonance is governed solely by the DC pump power. The following analysis elucidates this mechanism.

Beyond this typical resonance structure, our model identifies an additional phase-shift factor $1+i\gamma \sin\alpha \cdot M_{0}\xi \Gamma A_{0}/\left(\Gamma_{1}+\Gamma A_{0}\right)$ in Equation (6b). This factor arises from the superposition of two magnetic resonances with identical center frequency and linewidth but a 90° phase difference. The real part of this factor (unity) corresponds to the in-phase synchronous pumping resonance [22]. Its amplitude is proportional to $\cos\alpha \cdot \Gamma A_{-1}$, a term that represents the component of the modulated pumping rate perpendicular to the background field $B_{0}$. The imaginary part $i\gamma \sin\alpha \cdot M_{0}\xi \Gamma A_{0}/\left(\Gamma_{1}+\Gamma A_{0}\right)$ stems from a Radio-Frequency (RF)-excitation-like resonance [22] induced by the AC light-shift (equivalent to an oscillating fictitious field) interacting with the static $M_{z}$ polarization. This component has a 90° phase lag relative to the pump modulation, and its amplitude is jointly proportional to the AC light-shift magnitude ($\propto \cos\alpha \cdot M_{0}\xi \Gamma A_{-1}$) and the longitudinal polarization $M_{z}$ ($\propto \sin\alpha \cdot \Gamma A_{0}/\left(\Gamma_{1}+\Gamma A_{0}\right)$).

This leads to a key and counter-intuitive finding: despite being initiated by the AC light shift, the net phase shift is determined by the ratio of the two resonance amplitudes, which depends solely on the DC component of the pump rate, $\Gamma A_{0}$, not on its AC component, $\Gamma A_{-1}$. This independence arises because both amplitudes scale proportionally with $\Gamma A_{-1}$, consequently canceling out in their ratio. This crucial dependence is directly imprinted on the phase of the magnetic resonance signal.

### 3.3. Lineshapes of the Demodulated Signals

We now derive explicit expressions for the detected magnetic resonance signal from the steady-state solution for $M_{+}$ given by Equation (6b). The probe beam signal, denoted as Sig, is proportional to the component of the atomic magnetization along its propagation direction ($\hat{e_{y}}$), which is $M_{y}$. Since $M_{y}$ is the imaginary part of $M_{+}$, the probe signal can be formulated as shown in Equation (7), describing an oscillation at the pump modulation frequency ω:

(7) Sig=−K·My≈−K·Imcosα·M0Γ·A−1−iδω+Γ2+ΓA0(1+iγsinα·M0ξΓA0Γ1+Γ·A0)e−iωt,

in which *K* represents a proportional coefficient and Im(c) represents the imaginary part of the complex number *c*.

This signal is experimentally demodulated using a lock-in amplifier referenced at the frequency ω. The demodulated output is characterized by in-phase (*X*), quadrature (*Y*), magnitude (*R*), and phase (θ) components, as defined in Equation (8):

(8) $$\begin{array}{c} Sig=X\sin(\omega t)+Y\cos(\omega t)=R\sin(\omega t+\theta ). \end{array}$$

By equating this representation with the approximated analytical expression for Sig shown in Equation (7), we obtain the specific forms of these demodulated signals, which are summarized in the set of Equations:

(9a)X≈K⋅cosα ⋅ M0Γ ⋅ A−1δω2+Γ2+ΓA02⋅Γ2+ΓA0−δω ⋅ γsinα ⋅ M0ξΓA0Γ1+Γ ⋅ A0,(9b)Y≈−K⋅cosα ⋅ M0Γ ⋅ A−1δω2+Γ2+ΓA02⋅δω+Γ2+ΓA0 ⋅ γsinα ⋅ M0ξΓA0Γ1+Γ ⋅ A0,(9c)R≈K⋅cosα ⋅ M0Γ ⋅ A−1δω2+Γ2+ΓA02Γ1+Γ ⋅ A02+γsinα ⋅ M0ξΓA02Γ1+Γ ⋅ A0,(9d)θ≈arg1−iδωΓ2+ΓA0+arg1−iγsinα ⋅ M0ξΓA0Γ1+Γ ⋅ A0,

in which arg(c) represents the phase angle of the complex number *c*. These expressions exhibit characteristic magnetic resonance behavior. For instance, the amplitude *R* reaches its maximum under the resonance condition δω=0. Consequently, the variations of *X*, *Y*, *R*, and θ with the pumping modulation detuning δω are termed magnetic resonance curves, which display a Lorentzian lineshape.

**Figure 2:**
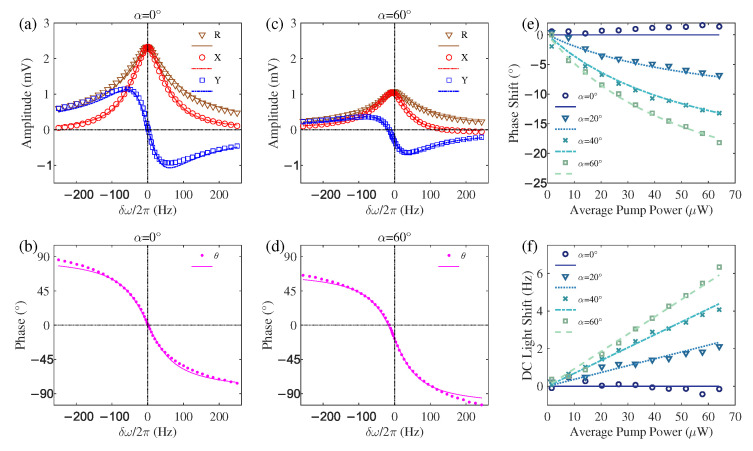
Dependence of magnetic resonance characteristics on orientation and pump power in a Bell–Bloom magnetometer. All data were acquired under a 50% duty cycle on-off modulation of the pump laser. Panels (**a**,**b**) show the demodulated amplitude (*R*), in-phase (*X*), quadrature (*Y*) signals, and phase (θ) of the magnetic resonance at an orientation of α=0°. Panels (**c**,**d**) present the corresponding signals at α=60°, where a significant phase shift is observed. Note: This phase shift originates from the AC light shift, a mechanism distinct from the orientation-dependent geometric effect reported in ref. [18]. The horizontal axis in (**a**–**d**) is the pump modulation detuning, defined as $\delta \omega =\omega -\gamma (B_{0}+{\bar{B}}_{\mathrm{LS}\|})$). The symbols in (**a**–**d**) represent experimentally recorded data, while the lines are fits to a Lorentzian lineshape. Panels (**e**,**f**) summarize the key parameters extracted from magnetic resonances under different orientations and pump powers. Specifically, panel (**e**) displays the phase shift, and panel (**f**) presents the DC light shift. The symbols in (**e**,**f**) denote the parameter values obtained from fitting the experimental magnetic resonance curves, and the lines are fits based on the theoretical model (see Equation (9)). The magnetic resonance curves shown in (**a**–**d**) were measured at a fixed average pump power of 64 µW.

A key insight comes from examining the phase expression θ in Equation (9d). Beyond the typical phase term $\arg\left(1-i\delta \omega /\left(\Gamma_{2}+\Gamma A_{0}\right)\right)$ arising from detuning, an additional phase shift $\arg\left(1-i\gamma \sin\alpha \cdot M_{0}\xi \Gamma A_{0}/\left(\Gamma_{1}+\Gamma A_{0}\right)\right)$ is present when the pump light is not perpendicular to the magnetic field (α≠0°). This phase shift causes the demodulated *X* and *Y* signals to deviate from their standard absorption and dispersion lineshapes, leading to distorted lineshapes. Since the phase shift itself is dependent on the pumping direction, the degree of distortion also varies with orientation, an example of which is shown in Figure 2a,c. When a phase-sensitive detection scheme is employed, for instance, using the zero-crossing of the *Y* signal to determine the Larmor frequency, this orientation-dependent distortion directly introduces a heading error in the measured magnetic field. It is vital to note that this heading error originates not from the well-known DC light shift, which changes the resonance frequency, but from the AC-light-shift-induced phase alteration of the magnetic resonance signal itself.

**Figure 3:**
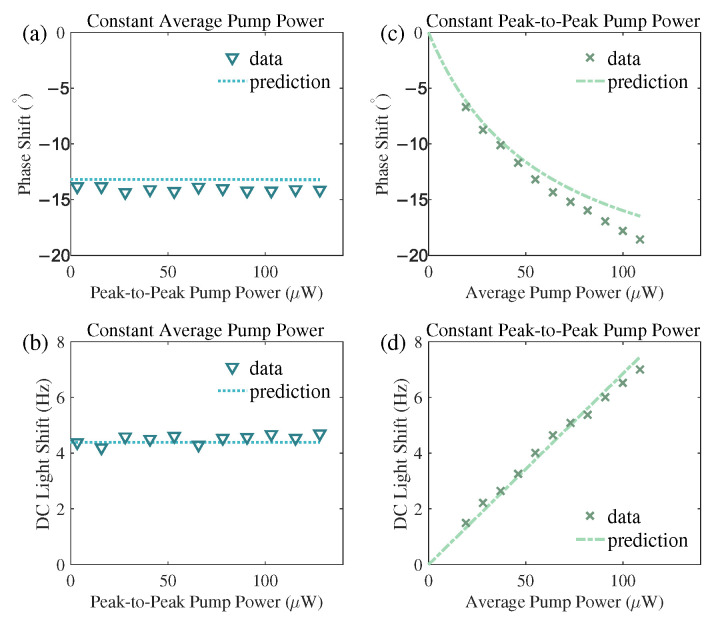
Dependencies of phase shift and DC light shift on AC and DC pump power. All measurements were performed at an orientation of α=40°. Panels (**a**,**b**) display the phase shift (**a**) and the DC light shift (**b**) as a function of the peak-to-peak pump power, while the average pump power is held constant at 64 µW. Panels (**c**,**d**) show the phase shift (**c**) and the DC light shift (**d**) as a function of the average pump power, while the peak-to-peak power is maintained at 38.4 µW. The symbols represent experimentally measured data. The solid lines are theoretical predictions calculated using the parameters derived from the global fits in Figure 2. The curves in (**a**,**c**) are based on the phase shift fit from Figure 2e, and the curves in (**b**,**d**) are based on the DC light shift fit from Figure 2f.

Since the phase shift induced by this AC light shift is a source of heading error, it is necessary to analyze its dependence on system parameters. Our investigation reveals that, despite originating physically from the AC light shift, the magnitude of this additional phase shift is determined solely by the average pump rate $\Gamma A_{0}$ and is independent of the modulated component $\Gamma A_{-1}$. This counter-intuitive dependence aligns perfectly with the physical interpretation derived from the steady-state $M_{+}$ solution given by Equation (6b) and is visually demonstrated in Figure 3a,c.

### 3.4. Model Validation and Justification of Approximations

The approximate analytical model derived above relies on the rotating-wave approximation and a first-order perturbation treatment of the light shift. Here, we provide a quantitative justification for these approximations using parameters from our experimental regime (Section 4).

The rotating-wave approximation is valid when the total relaxation rate is much smaller than the Larmor frequency. In our experiment, the maximum transverse relaxation rate $\Gamma_{2}+\Gamma A_{0}$ was 2π×67 Hz, while the Larmor frequency in the 1000 nT field was $\gamma B_{0}=2\pi \times 3500$ Hz. The ratio $\left(\Gamma_{2}+\Gamma A_{0}\right)/\left(\gamma B_{0}\right)\approx 0.019\ll 1$ confirms the excellence of this approximation.

The first-order treatment of the light shift requires $\gamma B_{\mathrm{LS}}/\left(\Gamma_{2}+\Gamma A_{0}\right)\ll 1$. Under our strongest pumping condition (α=40°), the maximum $\gamma B_{\mathrm{LS}}$ is estimated to be 2π×10.9 Hz, yielding a ratio of approximately 0.16 relative to the linewidth. This value, while larger than the rotating-wave approximation parameter, is sufficiently small to justify a first-order analysis.

Furthermore, we performed numerical simulations by directly integrating the full Bloch equations without these approximations. The excellent agreement between these numerical results and our analytical model, as will be shown in Section 5.3 alongside the experimental data, provides strong independent validation for our theoretical framework.

## 4. Results

To thoroughly investigate the influence of light shifts, particularly the phase shift in magnetic resonance curves induced by the AC light shift, we began by examining the effects of the pump-beam orientation and its average power under the commonly used on-off pump modulation scheme. The experiment was conducted under a background magnetic field of approximately 1000 nT. We changed the relative orientation between the pump laser and the magnetic field, varying the angle α from 0° to 60°. This corresponds to changing the angle between the pump propagation direction and the magnetic field from 90° to 30°. We also changed the average power of the pump beam, which was modulated with a 50% duty cycle on-off pattern, from 1.6 µW to 64 µW. For each specific combination of α and pump power, a magnetic resonance spectrum was acquired by sweeping the pump modulation frequency across a range of ±250 Hz around the Larmor frequency while recording the simultaneously demodulated *R*, *X*, *Y*, and θ signals.

Representative magnetic resonance curves obtained at a pump power of 64 µW for α=0° and α=60° are presented in Figure 2a–d, where the symbols correspond to experimental data and the lines are fits based on a Lorentzian model. The distorted lineshapes in Figure 2c,d reveal a phase shift in the magnetic resonance. It is crucial to distinguish this effect from a geometrically induced phase shift discussed in prior work [18]. Here, the phase shift arises from the AC light shift of the modulated pump beam. A key experimental distinction is that our probe beam was maintained perpendicular to the plane defined by the pump beam and the magnetic field, a configuration that suppresses the geometric phase shift mechanism of ref. [18]. The observations in Figure 2 therefore confirm the AC-light-shift-induced phase shift as a distinct and real effect. Analysis of these curves characterizes the specific signature of this new phase-shift mechanism. At α=0°, the demodulated *R* and *X* signals exhibit even symmetry about the resonance frequency, whereas *Y* and θ show odd symmetry, as expected in the absence of significant light shifts. In contrast, at α=60°, the AC light shift induces a distinct pattern: only the *R* signal maintains its even symmetry, while the lineshapes of *X* and *Y* become significantly distorted, and the θ curve exhibits a net downward shift. This specific distortion pattern is a direct consequence of the vector addition of the primary and secondary resonances described by our model (Equation (9)), serving as a fingerprint of the AC-light-shift mechanism. This phase shift would directly introduce a heading error in phase-sensitive detection schemes, for instance, when identifying the Larmor frequency from the zero-crossing point of the *Y* signal or θ signal.

The dependence of the magnetic resonance phase shift on the magnetometer orientation and pump power is systematically presented in Figure 2e. The data points represent the phase shifts extracted from experimental fits to the magnetic resonance curves, and the lines are global fitting results based on the additional phase shift term in Equation (9d). The specific fitting function used was $\arg(1-ia\sin\alpha \bar{P}/\left(b+\bar{P}\right))$, where *a* and *b* are free parameters and $\bar{P}$ is the average pump power. The global fitting yields parameters a=0.73±0.07 and b=65±10μW mean ± standard deviation). The relative uncertainties of approximately 10% for both parameters reflect the experimental precision of this calibration. The parameter *a* indicates that the Larmor frequency of the fictitious magnetic field induced by the pump light shift is about 0.73 times the pumping rate at a given optical power. The parameter *b* shows that the longitudinal relaxation rate $\Gamma_{1}$ is equivalent to the pumping rate produced by a 65 μW pump beam. Several key trends are evident from Figure 2e. At α=0°, increasing the pump power does not induce a significant phase shift. In contrast, for α>0°, the phase shift grows markedly with pump power, and a larger α angle leads to a more pronounced shift. This behavior is rooted in the physical mechanism of the phase shift. The shift originates from an RF-excitation-like magnetic resonance, which is itself induced by the interaction between the AC light shift and the longitudinal magnetization $M_{z}$. This resonance has a 90° phase difference relative to the primary synchronous optical pumping resonance. The phase of the combined signal is therefore determined by the amplitude ratio of these two resonances. Crucially, while both scale with the AC component of the pump rate, the amplitude of the RF-excitation-like resonance is also directly proportional to $M_{z}$. Consequently, the net phase shift is ultimately governed by the magnitude of $M_{z}$. A larger α angle allows the pump light to align more closely with the background field $B_{0}$, facilitating the buildup of a larger $M_{z}$ and thus a larger phase shift. Similarly, a higher average pump power also increases $M_{z}$, resulting in a larger phase shift. According to Equation (6a), $M_{z}$ is directly related to the DC component (average power) of the pump light and is independent of its AC component. This leads to the counter-intuitive phenomenon where the phase shift, though physically caused by the AC light shift, is quantitatively determined by the DC component of the pump light. Direct experimental evidence for this unique dependence will be presented in detail later in Figure 3a,c.

In addition to the phase shift induced by the AC light shift, the well-known DC light shift is another significant source of heading error, as it causes an orientation-dependent shift in the magnetic resonance frequency. It is important to note that the horizontal axis in the magnetic resonance spectra of Figure 2a–d is defined as the detuning $\delta \omega =\omega -\gamma (B_{0}+{\bar{B}}_{\mathrm{LS}\|})$. This definition inherently subtracts the frequency shift $\gamma {\bar{B}}_{\mathrm{LS}\|}$ caused by the DC light shift. Consequently, its effect is not directly visible in these resonance curves. Nevertheless, by fitting the magnetic resonance data, the influence of the DC light shift can be extracted from the variation of the fitted resonance frequency under different experimental conditions. The relationship between the extracted DC light shift $\gamma {\bar{B}}_{\mathrm{LS}\|}$ and both the orientation angle α and the average pump power $\bar{P}$ is summarized in Figure 2f. The symbols represent the DC light shift values obtained from the Lorentzian fits, and the lines are global fitting results based on the function $d\sin\alpha \cdot \bar{P}$. The fitting yields a parameter d=0.107±0.003Hz/μW. The results clearly demonstrate that the DC light shift is proportional to both sinα and the average pump power $\bar{P}$, indicating that a larger orientation angle α or a higher pump power leads to a more substantial DC light shift.

To experimentally verify the counter-intuitive prediction from our theoretical analysis, i.e., the AC-light-shift-induced phase shift depending on the DC component (average power) of the pump laser but not on its AC component, we employed a modified pump modulation scheme. The conventional on-off modulation, where the power switches between a constant value and zero, inherently couples the AC and DC components, making it impossible to separate their individual effects. For instance, in the 50% duty cycle modulation used for Figure 2, the average power is exactly half of the peak power. To decouple these components, we adopted a modulation where the pump power switches between two non-zero levels. To investigate the influence of the AC component, we varied the peak-to-peak power while maintaining a constant average power of 64 µW, as shown in Figure 3a. Conversely, to study the effect of the DC component, we changed the average power while keeping the peak-to-peak power fixed at 38.4 µW, with results presented in Figure 3c. Both experiments were conducted at α=40°. In both panels, the symbols represent the phase shift values obtained from fitting the experimentally recorded magnetic resonance spectra, and the lines are the theoretical predictions calculated using the parameters derived from Figure 2e (i.e., the function $\arg(1-ia\sin\alpha \bar{P}/\left(b+\bar{P}\right))$ with a=0.73±0.07 and b=65±10μW). The results clearly demonstrate that varying the peak-to-peak power has no significant impact on the phase shift. In contrast, increasing the average pump power leads to a marked increase in the phase shift magnitude, consistent with the theoretical prediction. The measured phase shifts align well with the theoretical predictions calculated using the parameters from Figure 2e. The slight systematic deviation, which becomes more noticeable at the highest power in Figure 3c, is within the expected range given the approximately 10% relative uncertainty in the fitting parameters *a* and *b*. This level of agreement, combined with the excellent consistency between the full numerical simulation and our model shown in Section 5.3, confirms that potential higher-order effects beyond our first-order approximation or minor parameter variations between independent experimental runs are not significant enough to affect our conclusions. Most importantly, the key experimental trends—the insensitivity to peak-to-peak power in Figure 3a and the clear dependence on average power in Figure 3c—are captured unambiguously and robustly by the theoretical model.

Under the same experimental configuration, we also examined the influence of the DC and AC components of the pump laser on the DC light shift, with the results presented in Figure 3b,d. The data in Figure 3b were acquired under the same conditions as Figure 3a, where the average pump power was held constant while its peak-to-peak power was varied. Similarly, the data in Figure 3d correspond to the conditions of Figure 3c, where the peak-to-peak power was fixed and the average power was changed. The symbols denote the DC light shift values obtained from fitting the experimental magnetic resonance spectra, and the lines represent the theoretical predictions calculated using the parameter established from Figure 2f, namely the relationship $d\sin\alpha \cdot \bar{P}$ with d=0.107±0.003Hz/μW. The results clearly demonstrate that the DC light shift is determined solely by the average pump power and is independent of the peak-to-peak power, which aligns well with the theoretical expectation.

## 5. DC Compensation Proposal

### 5.1. Core Concept of the DC Compensation Scheme

Theoretical modeling and experimental validation presented above confirm that the AC light shift of the modulated pump light in a Bell–Bloom magnetometer induces a phase shift in the magnetic resonance spectrum. Counter-intuitively, the magnitude of this shift is governed by the DC component of the pump power, not its AC component. As this phase shift depends on the magnetometer’s orientation, it introduces a heading error. Suppressing this shift is therefore crucial for enhancing measurement accuracy and is particularly beneficial for applications on moving platforms, such as aerial magnetic surveys or wearable bio-magnetic sensing. The data in Figure 3c demonstrate a direct correlation between the phase shift and the DC pump power. Theoretically, reducing the DC component of the pump light to zero would eliminate this specific phase shift and its associated heading error. However, for a modulated pump beam, setting the average power to zero is impractical, as it requires the pump light to be completely off, preventing the establishment of atomic polarization. Analysis of Equation (9d) reveals that the additional phase shift takes the form $\arg(1-i\gamma \sin\alpha \cdot M_{0}\xi \Gamma A_{0}/\left(\Gamma_{1}+\Gamma A_{0}\right))$. Achieving a net zero value for the product $M_{0}\xi \Gamma A_{0}$ through a specific configuration would similarly nullify the phase shift. It is known that reversing the handedness of the pump light’s circular polarization inverts the optical pumping target, changing $M_{0}$ to $-M_{0}$. This property suggests that a strategic combination of pump light polarizations could potentially cancel the $M_{0}\xi \Gamma A_{0}$ term, thereby providing a means to eliminate the associated phase shift at its source.

To mitigate the phase shift arising from the $M_{0}\xi \Gamma A_{0}$ term, existing compensation strategies primarily fall into two categories: spatial averaging and temporal averaging. Spatial averaging techniques involve either inserting a half-wave plate inside the vapor cell to flip the handedness of the circular polarization as the pump light propagates [16], or utilizing two spatially separated pump beams with identical power but opposite circular polarizations [13]. Both approaches suppress the phase shift in the magnetic resonance signal by averaging out the effect in the spatial domain. Alternatively, temporal averaging employs a push–pull pumping method, where the handedness of the pump light’s circular polarization is switched between two opposite states within each modulation cycle [17]. This ensures that the time-averaged value of $M_{0}\xi \Gamma A_{0}$ effectively vanishes. Regarding practical implementation, spatial averaging avoids the need for high-speed temporal polarization modulation of the pump beam. However, it requires integrating optical components inside the vapor cell or careful alignment of dual beams, posing greater engineering challenges for miniaturized sensors. The push–pull temporal averaging scheme, which utilizes a single pump beam with polarization modulation, offers a compact optical path [23]. This makes it attractive for applications where component count and optical alignment simplicity are paramount, such as wearable bio-magnetic sensing. However, its efficacy relies critically on the long-term stability of the polarization modulation, which can be influenced by environmental factors such as temperature fluctuations [16].

Building upon this understanding of the limitations associated with polarization stability, we propose an alternative DC compensation scheme. While it involves a primary modulated pump beam and a secondary continuous-wave beam, these beams are superimposed and share a common optical path, minimizing alignment complexity. The key distinction of this approach is that it shifts the technical challenge from high-speed polarization modulation to the control of optical power balance. The practical implications of this trade-off are discussed in Section 5.4.

**Figure 4:**
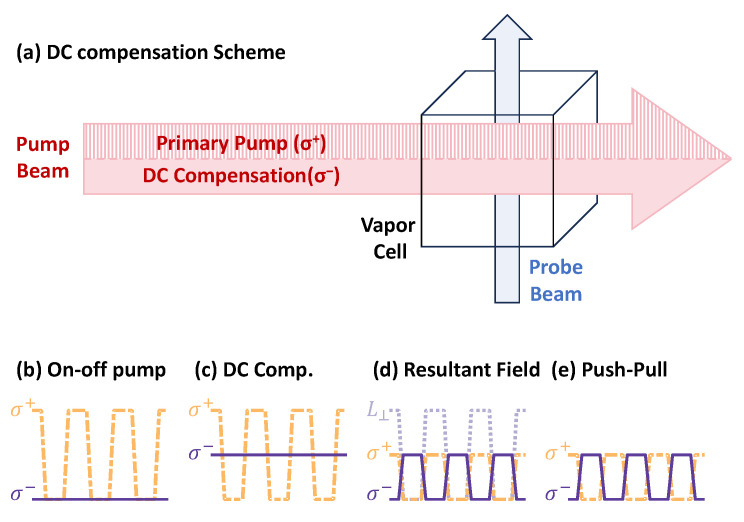
Proposed DC compensation scheme and analysis of the pumping field. (**a**) Schematic of the proposed DC light compensation scheme. A continuous compensation beam is superimposed on the primary modulated pump to suppress the AC-light-shift-induced phase shift. This compensation beam has the same average power but the opposite circular polarization. The primary beam ($\sigma^{+}$, dashed) and the compensation beam ($\sigma^{-}$, solid) are visually separated within the unified arrow for clarity but are co-propagating and spatially overlapped in the same optical path. (**b**–**e**) Timing diagrams and polarization analysis. (**b**,**c**,**e**) Optical pumping rates for the $\sigma^{+}$ (orange dashed line) and $\sigma^{-}$ (dark purple solid line) components for different schemes. (**b**) Standard on-off modulation. (**c**) The proposed DC compensation scheme. (**d**) Decomposition of the resultant optical field in the DC compensation scheme into its circularly polarized components ($\sigma^{+}$ and $\sigma^{-}$) and the newly generated linearly polarized component ($L_{⊥}$, light purple dotted line), whose intensity is twice that of the individual circular components. (**e**) Push–pull scheme.

As shown in Figure 4a, the core of our scheme is to introduce a continuous-wave compensation beam with opposite circular polarization that shares the same optical path as the primary modulated pump. The timing diagrams in Figure 4b–e elucidate how this configuration achieves compensation at a fundamental level. In the standard scheme (Figure 4b), only the modulated $\sigma^{+}$ pump is present, generating a non-zero DC pumping component that leads to a stable longitudinal magnetization $M_{z}$. It is precisely this $M_{z}$ that interacts with the AC light shift to produce the phase shift. Our DC compensation method (Figure 4c) addresses this root cause by adding a continuous $\sigma^{-}$ beam. The key is that its power is set to equal the average power of the $\sigma^{+}$ pump. This ensures the net DC optical pumping is canceled, which directly results in a net zero $M_{z}$. A decomposition of the resulting optical field in our scheme, shown in Figure 4d, provides further insight. It reveals that the net circular polarization indeed switches between $\sigma^{-}$ and $\sigma^{+}$, similar to a push–pull scheme. However, a key difference is the presence of a significant, amplitude-modulated linear polarization component during the pump phase. This linear component is a direct consequence of superimposing the two beams and has practical implications discussed in Section 5.4. With $M_{z}$ eliminated, the mechanism driving the AC-light-shift-induced phase shift is disabled. Simultaneously, the DC light shifts from the two counter-polarized beams also cancel each other out. Therefore, this approach simultaneously suppresses both sources of heading error. For comparison, the push–pull scheme (Figure 4e) also aims to cancel the net DC pumping, but it does so temporally by rapidly switching the polarization of a single beam between $\sigma^{+}$ and $\sigma^{-}$ within each cycle, resulting in a purely circular field without a linear component.

### 5.2. Theoretical Analysis and Validity

To theoretically validate the proposed compensation scheme, we introduce modifications to the Bloch equation (Equation (1)) that account for the contributions of the compensation beam. Within this theoretical framework, the compensatory DC beam is set to have the same optical power as the average power of the primary modulated pump beam but possesses the opposite circular polarization. Consequently, the pumping rate associated with the compensation beam is $\Gamma A_{0}$, and its optical pumping process drives the atomic spins toward a target polarization of $-M_{0}$.

The core physical idea of our compensation scheme is to achieve a net zero DC optical pumping effect. The primary modulated pump beam, with its DC component $\Gamma A_{0}$, polarizes the atoms along $+M_{0}$. The counter-polarized DC compensation beam, with an equal pumping rate $\Gamma A_{0}$, polarizes the atoms in the opposite direction, along $-M_{0}$. Ideally, these two opposing effects cancel out, resulting in no net DC polarization from the optical fields. This physical cancellation is mathematically embedded in the following modified Bloch equation:

(10) $$\begin{array}{c} \frac{dM}{dt}=-\gamma \left(B_{0}+B_{\mathrm{LS}}+B_{\mathrm{CLS}}\right)\times M-\left(\Gamma_{2}M_{x}\hat{e_{x}}+\Gamma_{2}M_{y}\hat{e_{y}}+\Gamma_{1}M_{z}\hat{e_{z}}\right)\\ -\Gamma_{P}\left(M-M_{0}\right)-\Gamma A_{0}\left(M+M_{0}\right), \end{array}$$

in which $B_{\mathrm{CLS}}=-M_{0}\Gamma A_{0}\xi$ is the light shift of the compensation beam.

To solve this modified system, we exploit its mathematical similarity to the original one. We define a set of effective parameters: $\Gamma_{2}^{\prime }=\Gamma_{2}+2\Gamma A_{0}$, $\Gamma_{1}^{\prime }=\Gamma_{1}+2\Gamma A_{0}$, and $\Gamma_{P}^{\prime }=\Gamma_{P}-\Gamma A_{0}$. Physically, $\Gamma_{2}^{\prime }$ and $\Gamma_{1}^{\prime }$ represent the total transverse and longitudinal relaxation rates, now enhanced by the additional pumping from both the primary and compensation beams. Crucially, $\Gamma_{P}^{\prime }$ represents the effective modulated pumping rate, as the DC components $\Gamma A_{0}$ from the primary beam and $-\Gamma A_{0}$ from the compensation beam cancel in the net DC pumping. Applying these substitutions transforms Equation (10) into a form identical to the original Bloch equation (Equation (1)):

(11) $$\begin{array}{c} \frac{dM}{dt}=-\gamma \left(B_{0}+B_{\mathrm{LS}}^{\prime }\right)\times M-\left(\Gamma_{2}^{\prime }M_{x}\hat{e_{x}}+\Gamma_{2}^{\prime }M_{y}\hat{e_{y}}+\Gamma_{1}^{\prime }M_{z}\hat{e_{z}}\right)-\Gamma_{P}^{\prime }\left(M-M_{0}\right), \end{array}$$

where $B_{\mathrm{LS}}^{\prime }=M_{0}\Gamma_{P}^{\prime }\xi$.

Since Equation (11) is formally identical to the original Bloch equation, its steady-state solution can be directly written down by applying the same effective parameter substitutions ($\Gamma_{2}\to \Gamma_{2}^{\prime }$, $\Gamma_{1}\to \Gamma_{1}^{\prime }$, and $\Gamma_{P}\to \Gamma_{P}^{\prime }$) to the original solution (Equation (6)):

(12a)Mz≈Mz(0)≈0,(12b)M+≈M+(−1)e−iωt≈cosα ⋅ M0Γ ⋅ A−1−iω−γB0+Γ2+2ΓA0e−iωt.

**Figure 5:**
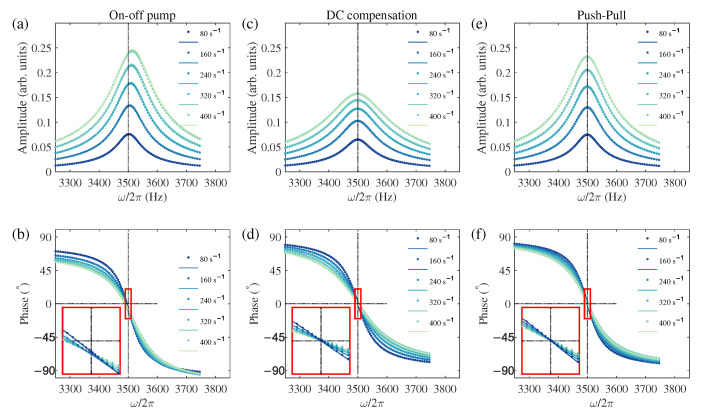
Numerical simulation of magnetic resonance signals under three pumping schemes at α=40°. Symbols: numerical simulation; Lines: theoretical predictions. (**a**,**b**) Standard on-off pumping shows significant light shifts: the resonance frequency and the phase curve shift as pump power increases (darker to lighter colors, representing the peak-to-peak pump rate from 80 s^−^^1^ to 400 s^−^^1^). (**c**,**d**) The proposed DC compensation scheme effectively suppresses both the frequency shift and the phase shift. (**e**,**f**) The push–pull scheme also eliminates these light-shift-induced errors and maintains a narrower linewidth than the DC scheme. These results validate that our DC compensation method achieves performance comparable to push–pull in suppressing heading errors. Insets in (**b**,**d**,**f**) show magnified views of the central region (the larger red box shows a magnified view of the area within the smaller central red box.), highlighting the behavior of the phase shift near resonance. The dotted black lines serve as guidelines to aid in identifying the trend of the data curve.

These results provide a clear theoretical validation. The zero longitudinal magnetization ($M_{z}\approx 0$) confirms the success of the physical cancellation of net DC polarization. The absence of light-shift-related terms in $M_{+}$ demonstrates the elimination of both the DC light shift (which would cause a frequency shift) and the AC-light-shift-induced phase shift. The only cost is a predictable increase in the magnetic resonance linewidth, from $\Gamma_{2}+\Gamma A_{0}$ to $\Gamma_{2}+2\Gamma A_{0}$, due to the additional optical pumping from the compensation beam. This broader linewidth directly results in a reduced peak amplitude of the magnetic resonance signal, as can be observed in the simulated amplitude *R* in Figure 5c. This signal reduction includes the depopulation effect of the non-circular light components on the atomic Zeeman sublevels. While a broader linewidth is a well-established trade-off against magnetometer sensitivity, this can be mitigated by systematic optimization of other parameters such as the vapor cell temperature, optical power, and optical frequency, or through techniques that operate beyond the standard quantum limit [7,24]. The result shown in Equation (12b) confirms the elimination of both the resonance frequency shift caused by the DC light shift and the phase shift induced by the AC light shift.

### 5.3. Numerical Verification

To validate our theoretical findings, we performed numerical simulations by directly solving the full Bloch equations without applying the rotating-wave approximation or first-order perturbation. The simulations used Equation (1) for the standard on-off scheme, Equation (10) for the DC compensation scheme, and Equation (A1) for the push–pull scheme. Parameters were chosen to match experimental conditions based on fits from Figure 2, with a magnetic field $B_{0}$ of 1000 nT, gyromagnetic ratio γ of 2π×3.5 Hz/nT, and relaxation rates $\Gamma_{1}$ and $\Gamma_{2}$ set to approximately 59 s^−^^1^ and 220 s^−^^1^ respectively. The parameter ξ was determined as 0.0334 nT·s. The complete procedure for determining these parameters from experimental data is provided in Appendix B. For fair comparison across schemes, the peak pump rate was used as a consistent power indicator. In the DC compensation case, the modulated pump’s peak rate matched that of the standard on-off scheme. For push–pull pumping, both $\sigma^{+}$ and $\sigma^{-}$ components were set at half the peak rate of the standard scheme, maintaining the same total pumping intensity. We varied the peak pump rate from 80 s^−^^1^ to 400 s^−^^1^, covering and extending beyond the experimental range in Figure 2 and Figure 3, corresponding to peak power of 88–440 µW or average power of 44–220 µW in on-off modulation.

The results are shown in Figure 5, which presents magnetic resonance spectra for the three schemes in two rows showing demodulated amplitude *R* and phase θ. The insets in Figure 5b,d,f provide a magnified view of the phase curves near resonance to better illustrate the presence or absence of a phase shift. Numerical data from the Runge-Kutta method appear as points, while theoretical predictions from Equations (6b), (12b), and (A3b) are shown as solid lines. Color shading represents the pump rate variation from 80 s^−^^1^ (darkest) to 400 s^−^^1^ (lightest). The standard scheme in Figure 5a,b shows clear DC light shift (frequency offset) and AC-light-shift-induced phase shift with increasing pump power. In contrast, both our DC compensation scheme (Figure 5c,d) and the push–pull scheme (Figure 5e,f) effectively suppress these effects, with push–pull maintaining the original linewidth and amplitude. The excellent agreement between simulation and theory confirms the validity of our analytical framework and the compensation schemes. Minor observed differences, such as a slight asymmetry in the simulated *R* signals at the highest pump rates, suggest the growing influence of higher-order light shift terms not captured by our first-order theoretical model. Critically, these minor discrepancies do not challenge the primary conclusion that both compensation schemes effectively suppress the light-shift-induced errors.

### 5.4. Discussion on Practical Considerations

While the DC compensation scheme offers a compelling approach to suppress light-shift-induced errors, its practical implementation requires careful consideration of several factors. These include the nature of the resulting optical field, the inherent performance trade-offs, its compatibility with specific atomic states such as the “stretched state”, and the stability requirements for the optical components.

A primary consideration is the polarization state of the superimposed optical field. The combination of the modulated $\sigma^{+}$ pump and the continuous $\sigma^{-}$ compensation beam results in a field that is, in general, elliptically polarized. This introduces a linear polarization component, which can optically pump atomic alignment. In a configuration where the two beams are perfectly coherent and in-phase, this could potentially generate alignment signals that interfere with the desired magnetic resonance. However, in a typical implementation where the modulated pump is frequency-shifted by an AOM, the two beams are not coherent. The resulting linear component’s azimuth rotates rapidly, effectively averaging the alignment to zero on the timescale of the atomic dynamics. Similarly, if the beams are derived from separate (incoherent) lasers, the relative phase fluctuates rapidly, achieving the same averaging effect. In both of these common scenarios, the fundamental ω-component magnetic resonance signal remains unaffected. It is also noted that the small AOM frequency shift (e.g., tens of MHz) has a negligible impact on the pumping rate due to the broad atomic transition (GHz-scale width broadened by buffer gas), thus preserving the compensation balance.

The scheme’s most significant performance trade-off is the inherent broadening of the magnetic resonance linewidth, as theoretically established in Section 5.2. While this was derived analytically, its practical implication is a direct trade-off between sensitivity and accuracy. The broader linewidth fundamentally limits the achievable magnetometric sensitivity, representing the price paid for suppressing the heading error. Consequently, this approach is particularly well-suited for applications where long-term operational stability is paramount and where the inherent sensitivity is sufficient for the target signals, or where it can be recovered by optimizing other system parameters, such as operating at a higher vapor cell temperature.

Furthermore, the scheme’s suitability depends on the atomic spin configuration. It is not recommended for magnetometers designed to operate with a “stretched state” [22,25]. In such systems, circularly polarized light pumps all atoms into a single dark state that does not interact with light. This dramatically narrows the magnetic resonance by reducing spin-exchange broadening. Any non-circular light in our scheme can destroy this dark state. This causes a severe signal loss, which is much worse than the simple linewidth broadening we discussed. Therefore, our scheme is better suited for conventional operating regimes that are less sensitive to this effect.

The efficacy of the compensation hinges on maintaining a precise balance between the average power of the primary pump and the power of the DC compensation beam. Any drift in the power of either beam, or in the modulation depth of the pump, will lead to a residual net DC pumping component, degrading the suppression of both the phase shift and the DC light shift. Consequently, the scheme requires precise control of optical power—a scalar quantity for which well-established feedback methods exist. Ensuring this power stability remains a key engineering requirement for robust long-term operation.

Finally, the scheme’s performance in high-density vapor cells, often used to enhance signal-to-noise ratio, warrants discussion. In such cells, strong absorption of the pump light can lead to a non-uniform power distribution along the beam path. If the primary and compensation beams experience different absorption rates due to their distinct polarization states and temporal profiles, the initial power balance will be disrupted along the cell, locally degrading the compensation efficiency. While the co-propagating nature of the beams mitigates this issue, this potential limitation should be considered for high-performance sensors operating at high atomic density.

### 5.5. Guidelines for Scheme Selection

The choice between these compensation schemes is a practical trade-off. The best option depends on the specific needs of the application. The push–pull scheme uses a single laser beam, which makes the optical setup compact. Besides, it does not cause extra resonance broadening beyond the usual effect of optical pumping, which helps in achieving high sensitivity. In comparison, our DC compensation scheme avoids the need for high-speed polarization modulation. However, it requires a careful balance of optical power between two beams, and it causes additional broadening of the resonance linewidth. Therefore, the push–pull scheme is a strong candidate when the priorities are a simple optical design and high sensitivity. The DC compensation scheme can be considered when the goal is to avoid polarization modulation, and when its trade-offs—such as resonance broadening and a more complex optical field—are acceptable.

## 6. Conclusions and Discussion

This study investigates the phase shift in magnetic resonance signals induced by the AC light shift in Bell–Bloom atomic magnetometers, a critical effect contributing to heading errors that limit their performance in high-precision mobile applications. Through theoretical modeling based on the Bloch equations and systematic experimental validation, we uncovered a counter-intuitive physical mechanism: although the phase shift is triggered by the AC light shift of the modulated pump laser, its magnitude is determined solely by the DC component (average power) of the pump laser and is independent of its AC modulation component. The underlying physics originates from an RF-excitation-like magnetic resonance, generated by the interaction between the AC light shift and the longitudinal atomic polarization, which has a 90° phase difference relative to the primary synchronous optical pumping resonance. Since the amplitude of the RF-excitation-like resonance depends on both the AC light shift strength and the longitudinal polarization $M_{z}$ established by the DC pump component, while the amplitude of the synchronous pumping resonance relies only on the AC component, their amplitude ratio and the consequent phase shift are governed exclusively by the DC pump component. Building on this mechanistic understanding, we proposed a novel DC compensation scheme. This approach involves superimposing a DC beam with opposite polarization and equal average power onto the original modulated pump light, physically canceling the net DC pumping effect and the DC light shift. Theoretically, this simultaneously eliminates both the AC-light-shift-induced phase shift and the DC light shift. A key feature of this scheme is that it avoids the need for high-speed polarization modulation, relying instead on the control of optical power. Furthermore, by merely adding a DC beam, it compensates for both sources of heading error. This co-propagation scheme, conducive to a compact design, is particularly promising for mobile sensing platforms, including uncrewed survey systems (aerial [26] and underwater [27]) and wearable biomagnetic sensors operating in unshielded environments [19,28,29].

Regarding the generality of our model, its framework extends beyond the specific square-wave amplitude modulation used in our experiments. The theoretical analysis relies on a Fourier decomposition of the pumping rate. For any periodic modulation waveform, such as sinusoidal or triangular, the physical distinction lies solely in the values of the Fourier coefficients. Since our derivation employs the rotating-wave approximation, which retains only the dominant DC and first-order harmonic components, the core results and the proposed compensation scheme remain valid. The model’s applicability is therefore broad and not restricted to a single modulation type.

Furthermore, the theoretical approach is adaptable to Bell–Bloom magnetometers employing other modulation parameters, such as polarization or laser frequency modulation [30,31]. This can be achieved by redefining the modulated quantity within the Bloch equations. For instance, polarization modulation would involve a periodic reversal of the optical pumping target vector, while frequency modulation would alter the pumping rate and the detuning-dependent light shift. Our model provides a versatile foundation for analyzing these variants by correspondingly adjusting the relevant parameters. As an example, we have applied this model to analyze the polarization-modulated push–pull scheme in Appendix A; the numerical simulation results are shown in Figure 5.

Concerning operation in different magnetic environments, our experiments were conducted at 1000 nT. The scheme’s performance in larger fields, such as the Earth’s field near 50 μT, warrants discussion. In such regimes, the nonlinear Zeeman effect may become non-negligible. It is noteworthy that our compensation scheme, by design, cancels the net DC polarization, a feature it shares with push–pull pumping methods which are known to mitigate NLZ-induced heading errors [17]. This suggests a potential resilience of our method against this effect, though a definitive confirmation would require further study with a density-matrix model in strong fields [32]. Regarding the nuclear Zeeman effect, our current probe laser configuration, locked to the F=3 to $F^{\prime }=4$ transition, already minimizes its influence by selectively sensing the F=4 ground-state spins. This strategy remains effective across a wide range of field strengths.

In summary, while this study successfully validates the core mechanism and compensation strategy, several avenues for future work remain to fully exploit its potential. Refining the theoretical model beyond the first-order approximation will be valuable for precisely characterizing performance under very high pump powers. In practice, however, stabilizing the pump laser frequency close to the atomic transition can substantially reduce the overall light shift, ensuring that the residual effects remain within the effective suppression range of our compensation scheme. Concurrently, the practical implementation will be evaluated in demanding scenarios, such as high-density vapor cells where differential absorption between the two beams could challenge the cancellation efficacy. Techniques to improve spin polarization uniformity, such as multi-pass cell designs, will be explored to strengthen robustness [33,34]. Beyond these refinements, operation in Earth-field conditions and the interaction with effects like the nonlinear Zeeman effect represent a significant and promising direction for applied research.

## Data Availability

All data needed to evaluate the conclusions in the paper are present in the paper. Additional data related to this paper may be requested from the authors.

## Appendix A. Modeling the Push–Pull Scheme

This appendix details the theoretical model for the push–pull pumping scheme. In this scheme, the pump polarization switches periodically between $\sigma^{+}$ and $\sigma^{-}$. To facilitate analysis and maintain a total pumping intensity comparable to the other schemes, the pumping process is treated as the superposition of two components.

The $\sigma^{+}$ component has a pumping rate of $\Gamma_{P\sigma^{+}}\left(t\right)=\Gamma_{P}\left(t\right)/2$ and drives the spins toward the target polarization $+M_{0}$. Conversely, the $\sigma^{-}$ component has a pumping rate of $\Gamma_{P\sigma^{-}}\left(t\right)=\Gamma_{P}\left(t-\pi /\omega \right)/2$ and drives the spins toward the opposite target $-M_{0}$.

The modified Bloch equation that includes the optical pumping and light shift from both components is given by

(A1) $$\begin{array}{c} \frac{dM}{dt}=-\gamma \left(B_{0}+B_{\mathrm{LS}\sigma^{+}}+B_{\mathrm{LS}\sigma^{-}}\right)\times M-\left(\Gamma_{2}M_{x}\hat{e_{x}}+\Gamma_{2}M_{y}\hat{e_{y}}+\Gamma_{1}M_{z}\hat{e_{z}}\right)\\ -\Gamma_{P\sigma^{+}}\left(M-M_{0}\right)-\Gamma_{P\sigma^{-}}\left(M+M_{0}\right), \end{array}$$

where $B_{\mathrm{LS}\sigma^{+}}=M_{0}\Gamma_{P\sigma^{+}}\xi$ and $B_{\mathrm{LS}\sigma^{-}}=-M_{0}\Gamma_{P\sigma^{-}}\xi$ are the fictitious magnetic fields arising from the light shift of the respective polarization component.

For the specific case of a 50% duty cycle square-wave modulation, the relationship $\Gamma_{P\sigma^{-}}\left(t\right)=\Gamma A_{0}-\Gamma_{P\sigma^{+}}\left(t\right)=\Gamma A_{0}-\Gamma_{P}\left(t\right)/2$ holds. Substituting this into the equation above allows it to be simplified to

(A2) $$\begin{array}{c} \frac{dM}{dt}=-\gamma \left(B_{0}+M_{0}\left(\Gamma_{P}-\Gamma A_{0}\right)\xi \right)\times M-\left(\Gamma_{2}M_{x}\hat{e_{x}}+\Gamma_{2}M_{y}\hat{e_{y}}+\Gamma_{1}M_{z}\hat{e_{z}}\right)\\ -\Gamma A_{0}M+\left(\Gamma_{P}-\Gamma A_{0}\right)M_{0}. \end{array}$$

Applying the rotating-wave approximation and a first-order perturbation analysis for the light shift to this simplified model yields the steady-state solutions for the magnetization

(A3a)Mz≈Mz(0)≈0,(A3b)M+≈M+(−1)e−iωt≈cosα ⋅ M0Γ ⋅ A−1−iω−γB0+Γ2+ΓA0e−iωt.

The solution shows that the longitudinal magnetization $M_{z}$ is zero, and the transverse magnetization $M_{+}$ precesses at the unshifted Larmor frequency $\gamma B_{0}$. This confirms that the push–pull scheme theoretically suppresses both the DC light shift (which causes a frequency shift) and the AC-light-shift-induced phase shift, without introducing additional resonance broadening beyond the inherent optical pumping contribution $\Gamma A_{0}$.

## Appendix B. Determination of Simulation Parameters

The parameters $\Gamma_{1}$, $\Gamma_{2}$, and ξ were extracted from global fits to the experimental data in Figure 2. Specifically:

- Parameter *a* from Figure 2e relates to the light shift, giving ξ=a/γ.
- Parameter *b* from the same figure and *d* from Figure 2f allow the calculation of the longitudinal relaxation rate via $\Gamma_{1}=2\pi db/a$.
- The transverse relaxation rate $\Gamma_{2}$ was obtained from the linewidth of magnetic resonance signals measured at low optical power.
